# The Antiproliferative Effect of Chakasaponins I and II, Floratheasaponin A, and Epigallocatechin 3-*O*-Gallate Isolated from *Camellia sinensis* on Human Digestive Tract Carcinoma Cell Lines

**DOI:** 10.3390/ijms17121979

**Published:** 2016-11-26

**Authors:** Niichiro Kitagawa, Toshio Morikawa, Chiaki Motai, Kiyofumi Ninomiya, Shuhei Okugawa, Ayaka Nishida, Masayuki Yoshikawa, Osamu Muraoka

**Affiliations:** 1 Pharmaceutical Research and Technology Institute, Kindai University, 3-4-1 Kowakae, Higashi-osaka, Osaka 577-8502, Japan; kitagawa@koshiroseiyaku.co.jp (N.K.); motai@koshiroseiyaku.co.jp (C.M.); ninomiya@phar.kindai.ac.jp (K.N.); okugawa_kameokanousan@koshiroseiyaku.co.jp (S.O.); 1311610173m@kindai.ac.jp (A.N.); m-yoshikawa@leto.eonet.ne.jp (M.Y.); 2 Koshiro Company Ltd., 2-5-8 Doshomachi, Chuo-ku, Osaka 541-0045, Japan; 3 Antiaging Center, Kindai University, 3-4-1 Kowakae, Higashi-osaka, Osaka 577-8502, Japan

**Keywords:** chakasaponin, floratheasaponin, (–)-epigallocatechin 3-*O*-gallate, anti-proliferative activity, tea flower, *Camellia sinensis*, apoptosis

## Abstract

Acylated oleanane-type triterpene saponins, namely chakasaponins I (**1**) and II (**2**), floratheasaponin A (**3**), and their analogs, together with catechins—including (–)-epigallocatechin 3-*O*-gallate (**4**), flavonoids, and caffeine—have been isolated as characteristic functional constituents from the extracts of “tea flower”, the flower buds of *Camellia sinensis* (Theaceae), which have common components with that of the leaf part. These isolates exhibited antiproliferative activities against human digestive tract carcinoma HSC-2, HSC-4, MKN-45, and Caco-2 cells. The antiproliferative activities of the saponins (**1**–**3**, IC_50_ = 4.4–14.1, 6.2–18.2, 4.5–17.3, and 19.3–40.6 µM, respectively) were more potent than those of catechins, flavonoids, and caffeine. To characterize the mechanisms of action of principal saponin constituents **1**–**3**, a flow cytometric analysis using annexin-V/7-aminoactinomycin D (7-AAD) double staining in HSC-2 cells was performed. The percentage of apoptotic cells increased in a concentration-dependent manner. DNA fragmentation and caspase-3/7 activation were also detected after 48 h. These results suggested that antiproliferative activities of **1**–**3** induce apoptotic cell death via activation of caspase-3/7.

## 1. Introduction

**Figure 1:**
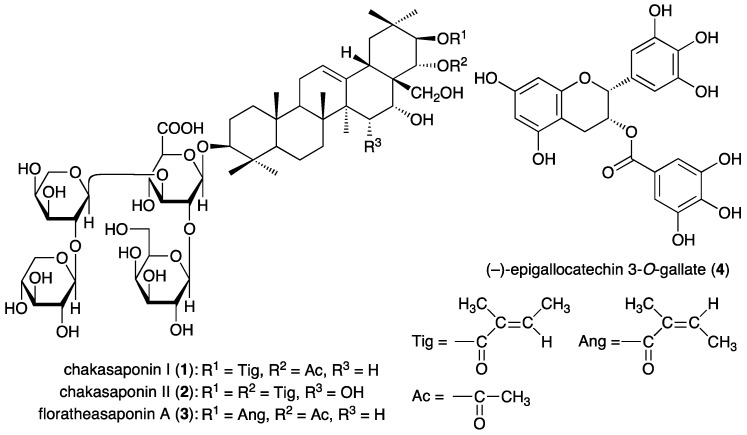
Structures of chakasaponins I (**1**) and II (**2**), floratheasaponin A (**3**), and (–)-epigallocatechin 3-*O*-gallate (**4**).

Saponins, which comprises a triterpene or steroid aglycone with oligosugar chains, are a large, structurally diverse group of bioactive natural products that are widely distributed in higher plants and marine organisms, such as starfish and sea cucumbers [1,2,3,4,5,6]. Saponins have been reported to possess a number of important bioactive properties, such as expectorant, anti-inflammatory, vasoprotective, hypocholesterolemic, immunomodulatory, hypoglycemic, cytotoxic, molluscicidal, antifungal, and antiparasitic activities [1,3,5,6]. Recently, we reported the identification and biological properties of saponin constituents from “tea flower”, the flower buds of *Camellia sinensis* (L.) O. Kuntze (Theaceae) [7,8,9,10,11]; the major saponin constituents were chakasaponins I (**1**) and II (**2**) and floratheasaponin A (**3**) (Figure 1). “Tea flower” has been used as food-garnishing agent in some Japanese dishes (e.g., botebote-cha in Shimane prefecture) or in drinks in some rural areas (e.g., batabata-cha in Niigata prefecture). With regard to the biofunctions of saponin constituents in “tea flower”, antihyperlipidemic, antihyperglycemic, gastroprotective, antiobesity, antiallergic, and pancreatic lipase and amyloid β aggregation-inhibitory effects have been reported [11]. A variety of health foods and beverages made from “tea flower” have been developed in Japan, Taiwan, and neighboring Asian countries based on the abovementioned evidence. In the current study, we investigated the antiproliferative activities of the active constituents in “tea flower”, namely saponins, catechins (including (–)-epigallocatechin 3-*O*-gallate (**4**)), flavonoids, and caffeine, which have components common with those of the leaf part (“green tea”) [12]. The antiproliferative effects against human digestive tract carcinoma HSC-2, HSC-4, MKN-45, and Caco-2 cells were examined.

## 2. Results and Discussion

### 2.1. Antiproliferative Activities of Constituents Isolated from “Tea Flower” against Human Gastric Carcinoma HSC-2, HSC-4, MKN-45, and Caco-2 Cells

Digestive tract carcinoma refers to a group malignancy located in the oral cavity, pharynx, larynx, esophagus, stomach, and large intestines and is the most common type of cancer worldwide [13]. In our previous studies of bioactive components from natural medicines isolated from the flowers of *Bellis perennis*, we observed antiproliferative activities of acylated oleanane-type triterpene saponins, perennisaponins A–T, against HSC-2, HSC-4, and MKN-45 cells [14]. The structures of perennisaponins resemble that of saponin constituents isolated from *C. sinensis*, and thus, similar activities were expected. Two analytical protocols have been developed with respect to nine acylated oleanane-type triterpene saponins, including chakasaponins I (**1**), II (**2**), and III, floratheasaponin A (**3**), and 15 polyphenols, including (+)-catechin, (–)-epicatechin, (–)-epigallocatechin, (–)-epicatechin 3-*O*-gallate, (–)-epigallocatechin 3-*O*-gallate (**4**), kaempferol, kaempferol 3-*O*-β-d-glucopyranoside, kaempferol 3-*O*-β-d-galactopyranoside, kaempferol 3-*O*-β-d-glucopyranosyl-(1→3)-α-l-rhamnopyranosyl-(1→6)-β-d-glucopyranoside, kaempferol 3-*O*-β-d-glucopyranosyl-(1→3)-α-l-rhamnopyranosyl-(1→6)-β-d-galactopyranoside, quercetin, quercetin 3-*O*-β-d-glucopyranoside, quercetin 3-*O*-β-d-galactopyranoside, rutin, and caffeine (Figure S1) [8,9,10]. Previously, the antiproliferative effects of dietary catechins and flavonoids from “green tea”, such as compound **4** and quercetin derivatives, against HSC-2, MKN-45, and Caco-2 cells have been reported [15,16,17,18]. To identify the active principles, the inhibitory activities of abovementioned “tea flower” constituents against human gastric carcinoma HSC-2, HSC-4, MKN-45, and Caco-2 cells were evaluated. As shown in Table 1, the saponin constituents (**1**–**3**) show antiproliferative activities, with IC_50_ values of 4.4–14.1 µM against HSC-2, 6.2–18.2 µM against HSC-4, 4.5–17.3 µM against MKN-45, and 19.3–40.6 µM against Caco-2 cells. The results are as follows: the IC_50_ values of chakasaponin I (**1**) against HSC-2, HSC-4, MKN-45, and Caco-2 cells were equal to 4.6, 17.5, 16.8, and 30.2 µM, respectively, and those of floratheasaponin A (**3**) were equal to 4.4, 6.2, 4.5, and 19.3 µM, respectively. The compounds having a common theasapogenol B (=barringtogenol C) moiety as an aglycone show relatively strong activities. These activities are higher than those of chakasaponin II (**2**), which has an IC_50_ value of 14.1, 18.2, 17.3, and 40.6 µM against HSC-2, HSC-4, MKN-45, and Caco-2 cells, respectively, and those of chakasaponin III, which has an IC_50_ value of 19.4, 22.1, 21.1, and 52.2 µM, respectively, except for the antiproliferative activity against MKN-45 cells. These results indicate that the presence of the 16*β*-hydroxy moiety in the aglycone part reduces the activity. Conversely, the polyphenol constituents (–)-epigallocatechin and (–)-epigallocatechin 3-*O*-gallate (**4**) show an antiproliferative activity against HSC-2 cells with IC_50_ values equal to 54.6 and 28.3 µM, respectively, whereas (–)-epigallocatechin, compound **4**, quercetin, and quercetin 3-*O*-β-d-glucopyranoside have IC_50_ values of 23.8, 27.2, 77.2, and 67.3 µM, respectively, against HSC-4 cells (Table S1). The concentration dependencies of the antiproliferative activity of compounds **1**–**4** against HSC-2 cells are observed in a range of 3–100 µM for compounds **1**, **2**, and **4** and a range of 0.3–10 µM for compound **3**, as shown in Figure S2.

### 2.2. Effects of Cell Cycle Distribution in HSC-2 Cells

**Figure 2:**
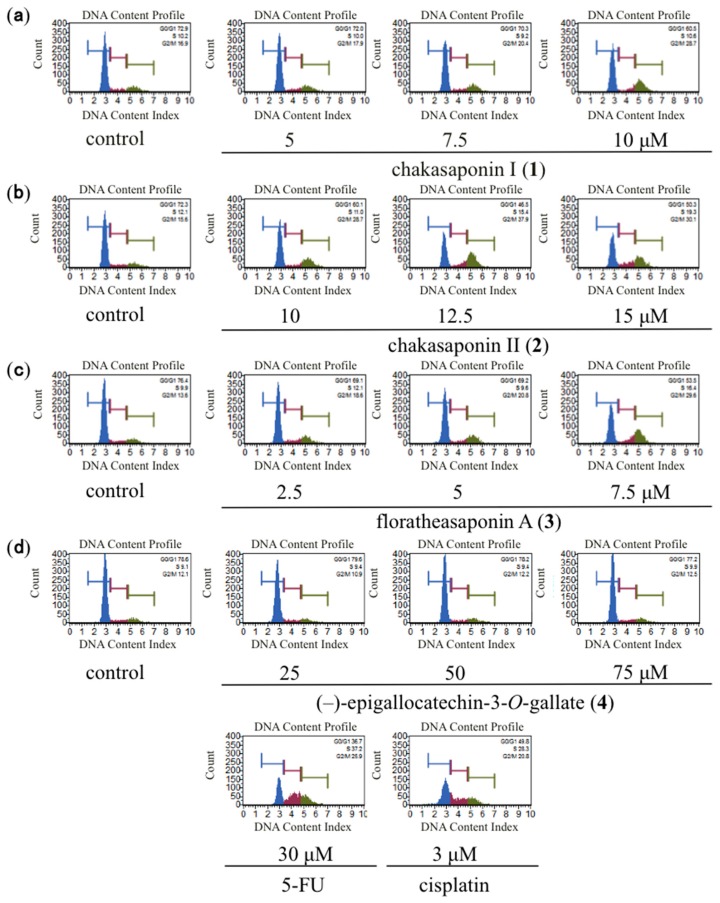
Effects of **1**–**4**, 5-FU, and cisplatin on the cell cycle distribution of HSC-2 cells. Cell cycle distribution was measured by Muse^®^ Cell Analyzer using a Muse Cell Cycle Kit (Merck Millipore, Darmstadt, Germany); HSC-2 cells were treated with (**a**) 5, 7.5, and 10 µM of **1**; (**b**) 10, 12.5, and 15 µM of **2**; (**c**) 2.5, 5, and 7.5 µM of **3**; (**d**) 25, 50, and 75 µM of **4**, 30 µM of 5-FU, and 3 µM of cisplatin for 48 h; the data represent the mean percentages ± SD of total apoptosis (*n* = 3); commercial 5-FU and cisplatin were purchased from Wako Pure Chemical Industries, Ltd. (Osaka, Japan).

Apoptosis and cell cycle dysfunction are closely associated biochemical processes, and any disturbance in cell cycle progression may lead to apoptotic cell death [19]. The effects of cell cycle distribution were analyzed to determine the mechanism associated with the growth inhibitory effect of chakasaponins I (**1**) and II (**2**), floratheasaponin A (**3**), and (–)-epigallocatechin 3-*O*-gallate (**4**) on HSC-2 cells [20,21]. The cell distribution in G0/G1, S, and G2/M phases shown in blue, red, and green areas, respectively, were determined after a 48 h incubation (Figure 2). As shown in Table 2, compounds **1**–**3** significantly induce populations of the cell distribution in S and G2/M phases, but reduce that of the G0/G1 phase. However, compound **4** does not affect the cell cycle distribution at the effective concentration. These results imply that compounds **1**–**3** induced the cell cycle arrest at the G2/M phase.

### 2.3. Quantification of Apoptotic Cell Death Using Annexin-V Binding Assay in HSC-2 Cells

**Figure 3:**
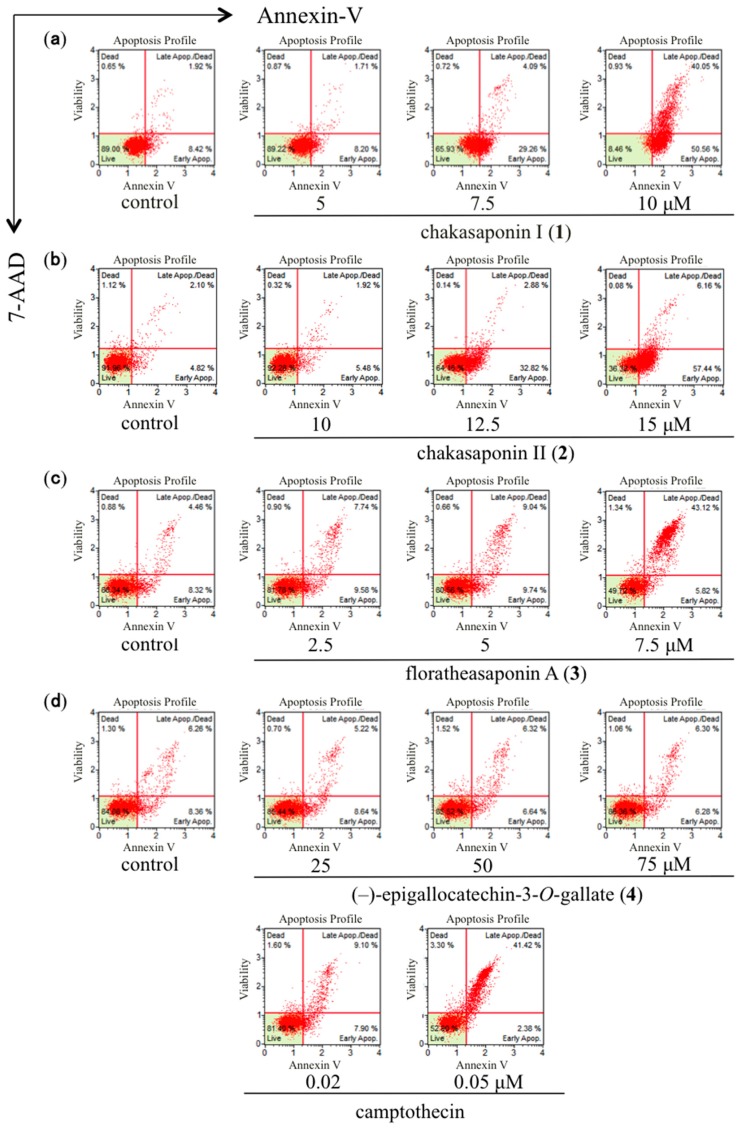
Effects of **1**–**4** and camptothecin on the apoptosis of HSC-2 cells. Annexin-V-binding was measured by Muse^®^ Cell Analyzer using a Muse Annexin-V and Deal Cell Kit (Merck Millipore); HSC-2 cells were treated with (**a**) 5, 7.5, and 10 µM of **1**; (**b**) 10, 12.5, and 15 µM of **2**; (**c**) 2.5, 5, and 7.5 µM of **3**; (**d**) 25, 50, and 75 µM of **4** and 0.02 and 0.05 µM of camptothecin for 24 h; the data represent the mean percentages ± SD of total apoptosis (*n* = 3); commercial camptothecin was purchased from Wako Pure Chemical Industries, Ltd. (Osaka, Japan).

Apoptosis plays an important role in the homeostatic maintenance of the tissue by selectively eliminating excessive cells. On the other hand, the induction of apoptosis of carcinoma cells is also recognized to be useful in cancer treatment, since cytotoxic agents used in chemotherapy of leukemia and solid tumors are known to cause apoptosis in target cells. Thus, the induction of apoptosis of cancer cells may be useful in cancer treatment [22]. To determine the apoptosis-inducing effects of compounds **1**–**4** against HSC-2 cells, staining with annexin-V/7-aminoactinomycin D (7-AAD) as a marker of early and late apoptotic events was performed (Figure 3) [23]. As shown in Table 3, the percentage of total apoptosis of compounds **1**–**3** increase in a concentration-dependent manner. However, compound **4** does not induce apoptotic cell death by this annexin-V-binding assay.

### 2.4. Evaluation of Apoptotic Morphological Changes in HSC-2 Cells

**Figure 4:**
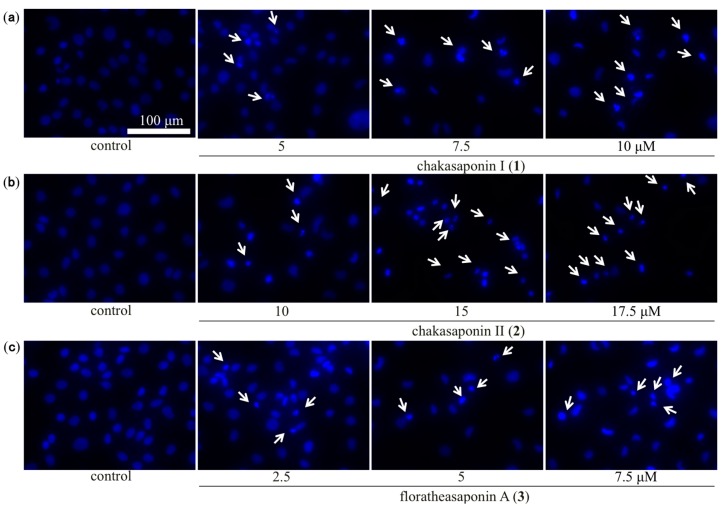

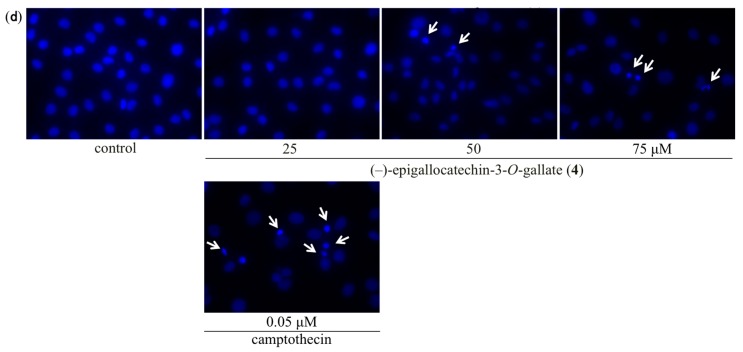
Morphological analysis of HSC-2 cells treated with **1**–**4** and camptothecin. Morphology of representative fields of HSC-2 cells stained with 4′,6-diamidino-2-phenylindole dihydrochloride (DAPI) after treatment with (**a**) 5, 7.5, and 10 µM of **1**; (**b**) 10, 15, and 17.5 µM of **2**; (**c**) 2.5, 5, and 7.5 µM of **3**; (**d**) 25, 50, and 75 µM of **4** and 0.05 µM of camptothecin for 48 h; the cells indicated by *arrows* represent fragmented and condensed nuclear chromatins; commercial camptothecin was purchased from Wako Pure Chemical Industries, Ltd. (Osaka, Japan).

Representative morphological features of apoptosis in HSC-2 cells were examined under an inverted light fluorescence microscope using 4′,6-diamidino-2-phenylindole dihydrochloride (DAPI) as a staining agent [24]. As shown in Figure 4, the cell shrinkage caused by compounds **1**–**3** was mediated partially through apoptosis induction as nuclear chromatin condensation. Nuclei of the cells treated with compound **4** showed the obvious changes only slightly, while evident nuclear condensation was observed in cells treated with compounds **1**–**3**.

### 2.5. DNA Fragmentation in HSC-2 Cells

**Figure 5:**
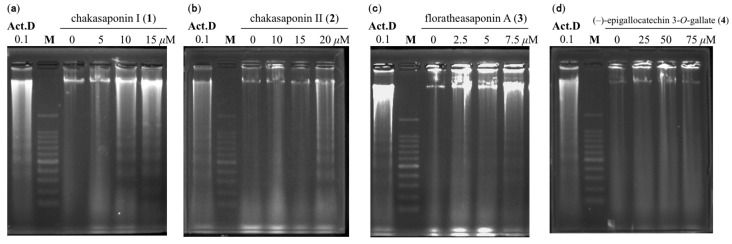
DNA fragmentation in HSC-2 cells treated with **1**–**4** and actinomycin D. Representative DNA fragmentation of HSC-2 treated with (**a**) 5, 10, and 15 µM of **1**; (**b**) 10, 15, and 20 µM of **2**; (**c**) 2.5, 5, and 7.5 µM of **3**; (**d**) 25, 50, and 75 µM of **4**, and (Act.D) 0.1 µM of actinomycin D for 48 h; (M) represents a marker (100 bp DNA ladder); commercial actinomycin D was purchased from Wako Pure Chemical Industries, Ltd. (Osaka, Japan).

DNA fragmentation in HSC-2 cells treated with compounds **1**–**4** were examined. Apoptotic cells display condensed chromatin and fragmented nuclei, but nonapoptotic cells maintain their structure [24,25,26]. As shown in Figure 5, DNA ladder formation, which is indicative of apoptosis, is observed in HSC-2 cells treated with compounds **1**–**3** for 48 h at effective concentrations. This fragmentation shows the same pattern as those treated with actinomycin D at a concentration of 0.1 µM.

### 2.6. Effects of Caspase-3/7 in HSC-2 Cells

**Figure 6:**
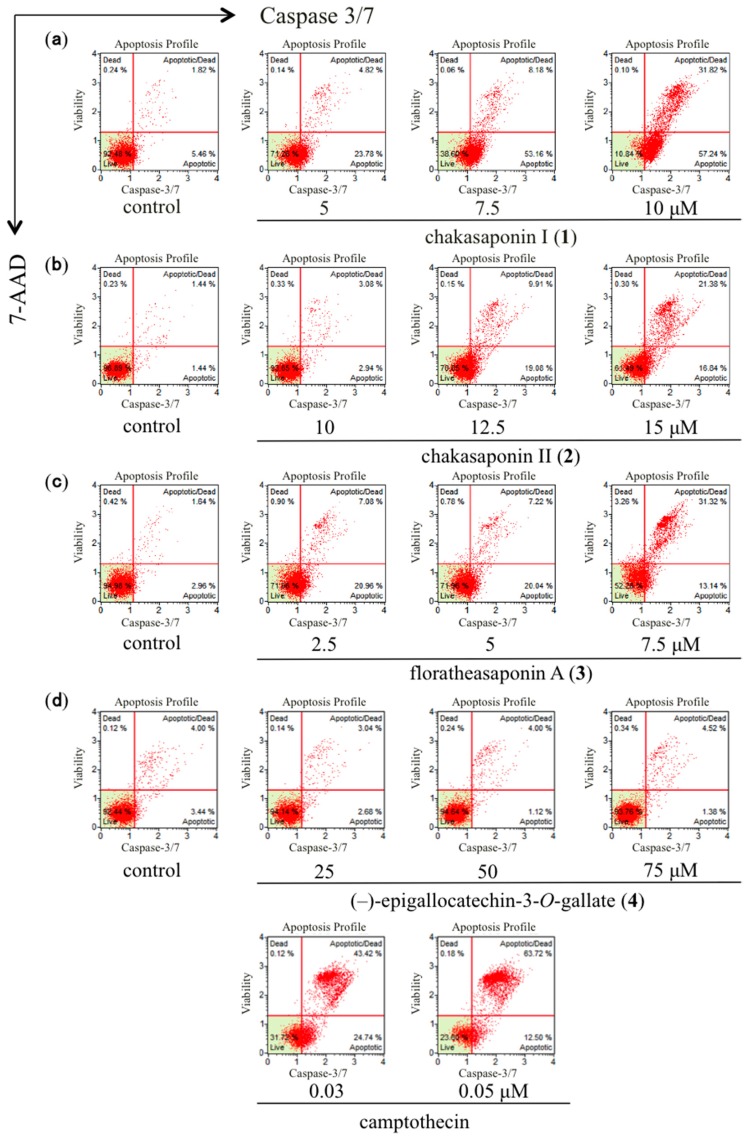
Effects of **1**–**4** and camptothecin on caspase-3/7 in HSC-2 cells. Activities of caspase-3/7 were measured by Muse^®^ Cell Analyzer using a Muse Caspase-3/7 Kit (Merck Millipore); HSC-2 cells were treated with (**a**) 5, 7.5, and 10 µM of **1**; (**b**) 10, 12.5, and 15 µM of **2**; (**c**) 2.5, 5, and 7.5 µM of **3**; (**d**) 25, 50, and 75 µM of **4** and 0.03 and 0.05 µM of camptothecin for 24 h; the data represent the mean percentages ± SD of total apoptosis (*n* = 3); commercial camptothecin was purchased from Wako Pure Chemical Industries, Ltd. (Osaka, Japan).

Caspase play a central role in the apoptotic signaling. During apoptosis, caspase activity contributes to the degradation of DNA and leads to further disruption of cellular components, resulting in alterations of cell morphology [22,27,28,29]. To confirm that caspases are involved the enzyme activity of caspase-3/7 in HSC-2 cells after coculture with compounds **1**–**4**, the activities of caspase-3/7 were measured by Muse^®^ Cell Analyzer (Figure 6). As shown in Table 4, the activation of caspase-3/7 by compounds **1**–**3** was found to occur in a concentration-dependent manner. These results suggest that the antiproliferative effects of acylated saponin constituents isolated from “tea flower” (**1**–**3**) against HSC-2 involve apoptotic cell death via activation of caspase-3/7. The efficacies of these saponins were found to be stronger than that of compound **4**, the most abundant polyphenol constituent in “green tea”.

## 3. Materials and Methods

### 3.1. Chemicals Constituents from “Tea Flower”

In previous studies, chakasaponins I (**1**, 0.497%), II (**2**, 0.153%), and III (0.194%), floratheasaponin A (**3**, 0.038%), (–)-epicatechin 3-*O*-gallate (0.015%), kaempferol (0.00080%), kaempferol 3-*O*-β-d-glucopyranoside (0.021%), kaempferol 3-*O*-β-d-galactopyranoside (0.011%), kaempferol 3-*O*-rutinoside (0.051%, 0.042%), kaempferol 3-*O*-β-d-glucopyranosyl-(1→3)-α-L-rhamnopyranosyl-(1→6)-β-d-glucopyranoside (0.409%), kaempferol 3-*O*-β-d-glucopyranosyl-(1→3)-α-L-rhamnopyranosyl-(1→6)-β-d-galactopyranoside (0.047%), quercetin 3-*O*-β-d-glucopyranoside (0.0063%), quercetin 3-*O*-β-d-galactopyranoside (0.0032%), and rutin (0.021%), were obtained from the methanol extract form the dried flower buds of *C. sinensis* collected in Fujian province, China (CSS-F1) [9]. The other chemicals, (–)-epigallocatechin 3-*O*-gallate (**4**), (+)-catechin, (–)-epicatechin, (–)-epigallocatechin, (–)-epicatechin 3-*O*-gallate, and quercetin were purchased from Funakoshi Co., Ltd. (Tokyo, Japan) and caffeine was from Nakalai Tesque Inc. (Kyoto, Japan) (Figure S1).

### 3.2. Reagents

Fetal bovine serum (FBS) was purchased from Life Technologies (Carlsbad, CA, USA); minimum essential medium (MEM) and RPMI 1640 medium were from Sigma-Aldrich Chemical (St. Louis, MO, USA); other chemicals were from Wako Pure Chemical Industries, Co., Ltd. (Osaka, Japan). The 96-well microplate was purchased from Sumitomo Bakelite Co., Ltd. (Tokyo, Japan).

### 3.3. Cell Viability Assays

HSC-2 (RCB1945), HSC-4 (RCB1902), MKN-45 (RCB1001), and Caco-2 cells (RCB0988) were obtained from RIKEN Bio Resource Center (Thukuba, Japan). Cells were cultured in MEM (for HSC-2 and Caco-2) or RPMI 1640 medium (for HSC-4 and MKN-45) containing FBS (10% for HSC-2, HSC-4, and MKN-45; 20% for Caco-2), 0.1 mM nonessential amino acids (for Caco-2), penicillin G (100 U/mL), and streptomycin (100 µg/mL) at 37 °C under a 5% CO_2_ atmosphere. The cells were inoculated into a 96-well tissue culture plate (HSC-2 and HSC-4: 3 × 10^3^; MKN-45: 7.5 × 10^3^ cells/well; Caco-2: 2 × 10^4^ cells/well in 100 µL/well). After 24 h incubation, 100 μL/well of medium containing a test sample was added. After 48 h incubation, cell viability was assessed using the 3-(4,5-dimethylthiazol-2-yl)-2,5-diphenyltetrazolium bromide (MTT) colorimetric assay. In this assay, 20 µL of MTT (5 mg/mL in phosphate-buffered saline (PBS(–)) solution was added to the medium. After 4 h incubation, the medium was removed, and 100 µL of isopropanol containing 0.04 M HCl was added to dissolve the formazan produced in the cells. The optical density (OD) of the formazan solution was measured using a microplate reader at 570 nm (reference: 655 nm). Inhibition (%) was obtained by the following formula and the IC_50_ was determined graphically.

Inhibition(%)=((O.D.(sample)−O.D.(control))/O.D.(control))×100

Each test compound was dissolved in dimethylsulfoxide (DMSO), and this solution was added to the medium (final DMSO concentration: 0.5%). Cisplatin, 5-fluorouracil (5-FU), doxorubicin, camptothecin, and taxol were used as reference compounds.

### 3.4. Cell Cycle Analysis

The cell cycle distribution analysis was measured by Muse^®^ Cell Analyzer (Merck-Millipore, Darmstadt, Germany) using a Muse Cell Cycle Assay Kit (Merck-Millipore) according to the manufacturer’s instructions. Briefly, HSC-2 cells were inoculated into a 6-well tissue culture plate (2 × 10^5^ cells/1.5 mL/well) and cultured in MEM containing 10% FBS, penicillin G (100 U/mL), and streptomycin (100 µg/mL) at 37 °C under a 5% CO_2_ atmosphere. After 24 h incubation, 500 µL/well of medium containing the test sample was added. After 48 h treatment, the cells were harvested by trypsinization, and suspended in 300 µL of PBS(–). The cells were added in 700 µL of ice-cold ethanol and incubated overnight at −20 °C. The fixed cells were collected by centrifugation at 300× *g* for 5 min and washed twice with 250 µL of PBS(–), then suspended in 200 µL of cell cycle reagent and incubated for 30 min in dark [30]. Each test compound was dissolved in DMSO, and the solution was added to the medium (final DMSO concentration: 0.5%). 5-FU was used as a reference compound.

### 3.5. Annexin-V/7-AAD Assay

Apoptosis was measured by a Muse Annexin-V and Dead Cell Kit (Merck-Millipore) according to the manufacturer’s instructions. Briefly, HSC-2 cells were inoculated into a 6-well tissue culture plate (2 × 10^5^ cells/1.5 mL/well). After 24 h incubation, 500 µL/well of medium containing the test sample was added. After 24 h treatment, the cells were harvested by trypsinization and a 100 µL cell suspension was labeled for 20 min in the dark with the same volume of Muse Annexin-V and Dead Cell Reagent. Subsequently, quantitative detection of annexin-V/7-AAD-positive cells was performed using the Muse^®^ Cell Analyzer. Cells stained with annexin-V only were defined as early apoptotic, while annexin-V and 7-AAD double-stained cells were defined as late apoptotic [14]. Each test compound was dissolved in DMSO, and the solution was added to the medium (final DMSO concentration: 0.5%). Camptothecin was used as a reference compound.

### 3.6. DAPI Staining for Morphological Analysis

HSC-2 cells (2 × 10^5^ cells/2 mL/well) were seeded onto coverslips in a 6-well tissue culture plate and cultured in MEM containing 10% FBS, penicillin G (100 U/mL), and streptomycin (100 µg/mL) at 37 °C under 5% CO_2_ atmosphere. After 24 h incubation, the medium was replaced with 2 mL fresh medium per well containing the test sample; then, the cells were cultured for 48 h. Next, the medium was removed and washed twice with PBS(–) and fixed with 4% paraformaldehyde phosphate buffer solution (pH 7.4, Wako Pure Chemical Industries, Ltd., Osaka, Japan). The cells were permeabilized by 0.2% Triton X-100 in PBS(–), stained with DAPI (1 µg/mL in PBS(–)), and were observed by fluorescence microscopy (EVOS^®^ FL Cell Imaging System, Thermo Fisher Scientific, Waltham, MA, USA) [14]. Each test compound was dissolved in DMSO, and the solution was added to the medium (final DMSO concentration: 0.5%). Camptothecin was used as a reference compound.

### 3.7. Agarose Gel Electrophoresis for the Detection of DNA Fragmentation

HSC-2 cells were inoculated into a 6-well tissue culture plate (2 × 10^5^ cells/1.5 mL/well). After 24 h incubation, 500 µL/well of medium containing the test sample was added. After 48 h treatment, the cells were harvested and suspended in a solution containing 50 mM Tris-HCl (pH 8.0), 150 mM NaCl, 10 mM ethylenediaminetetraacetic acid (EDTA), and 0.5% sodium dodecyl sulfate (SDS) at room temperature for 30 min. The lysates were incubated with RNase A (100 µg/mL) for 1 h at 37 °C, then proteinase K (500 µg/mL) was added and incubated at 50 °C for 2 h. DNA was extracted twice with an equal volume of 25:24:1 (*v*/*v*/*v*) phenol:chloroform:isoamyl alcohol and was purified by ethanol precipitation. The DNA was subjected to electrophoresis on 2.0% agarose gels and stained by ethidium bromide. The DNA bands were detected under UV illumination [24]. Each test compound was dissolved in DMSO, and the solution was added to the medium (final DMSO concentration: 0.5%). Actinomycin D was used as a reference compound.

### 3.8. Caspase-3/7 Assay

Apoptotic status based on caspase-3/7 activation was measured by a Muse Caspase-3/7 Kit (Merck-Millipore). Briefly, HSC-2 cells were inoculated into a 6-well tissue culture plate (2 × 10^5^ cells/1.5 mL/well). After 24 h incubation, 500 μL/well of medium containing the test sample was added. After 48 h treatment, the cells were harvested by trypsinization and stained according to the manufacturer's instructions. Subsequently, quantitative detection of caspase-3/7-positive cells was performed using the Muse^®^ Cell Analyzer [31,32,33]. Each test compound was dissolved in DMSO, and the solution was added to the medium (final DMSO concentration: 0.5%). Camptothecin was used as a reference compound.

### 3.9. Statistics

Values are expressed as means ± S.E.M. Significant differences were calculated using Dunnett’s test. Probability (*p*) values less than 0.05 were considered significant.

## 4. Conclusions

Acylated oleanane-type triterpene saponins obtained from the flower buds of *C. sinensis* (tea flower), namely chakasaponins I (**1**) and II (**2**) and floratheasaponin A (**3**), and the most abundant polyphenol constituent in “green tea”, (–)-epigallocatechin 3-*O*-gallate (**4**), showed antiproliferative activities against human digestive tract carcinoma HSC-2, HSC-4, MKN-45, and Caco-2 cells. These activities of the saponins (**1**–**3**, IC_50_ = 4.4–14.1, 6.2–18.2, 4.5–17.3, and 19.3–40.6 µM, respectively) were more potent than those of **4** (IC_50_ = 28.3, 27.2, >100, and >100 µM, respectively). In our previous study, we have demonstrated that a similar acylated oleanane-type triterpene saponin obtained from the flowers of *Bellis perennis* (Asteraceae), perennisaponin O, showed a relatively strong activity against HSC-2, HSC-4, and MKN-45 cells (IC_50_ = 11.2, 14.3, and 6.9 µM, respectively). Furthermore, the mechanism of action of perennisaponin O against HSC-2 was found to involve apoptotic cell death [14]. To clarify the mechanisms of action of compounds **1**–**3** against HSC-2 cells, effects of cell cycle distribution were analyzed at each effective concentration. The present study demonstrated a significant inhibition of cell proliferation by compounds **1**–**3** with cell cycle arrest occurring at the G2/M phase in a concentration-dependent manner. These saponins (compounds **1**–**3**) efficiently induce apoptosis in HSC-2 cells, as demonstrated by annexin V/7-AAD assay, morphological changes, and DNA fragmentation. Furthermore, the apoptotic cell death triggered by compounds **1**–**3** in HCS-2 cells was dependent on the activation of caspase-3/7. The efficacy of these saponins (**1**–**3**) as apoptosis inducers was found to be higher than that of compound **4**. Recently, compound **4** has received great attention in cancer research related to cancer preventive effects [34,35,36], the synergistic enhancement by the combination with different anticancer drugs [37,38], and clinical applications [39,40,41]. It is well recognized that compound **4** is able to bind to multiple molecular targets, thus, it can affect a range of signaling pathways, resulting in growth inhibition, apoptosis or suppressions of invasion, angiogenesis, and metastasis [42]. Ability of compound **4** to induce cell death in cancer cells is considered a key mechanism related to its anticancer function [43]. There are conflicting reports of apoptotic and nonapoptotic cell death induced by compound **4**, and the exact molecular mechanisms have not been fully elucidated yet. However, most of the previous reports have concluded that this compound induces caspase-mediated apoptosis in various tumor cells via the mitochondrial pathway [44,45] or via the death receptor [46,47,48]. Conversely, several reports demonstrated the involvement of nonapoptotic cell death, such as caspase-independent necrosis-like cell death [49] or reactive oxygen species (ROS)-mediated lysosomal membrane permeabilization [50]. The detailed mechanism of action of the antiproliferative activity of compound **4** needs further studies. In conclusion, we herein described that the saponin constituents of “tea flower” compounds **1**–**3** showed some antiproliferative activities against HSC-2 cells by the apoptotic pathway via caspase-3/7 activation. On the basis of the abovementioned evidence, these saponins can potentially be useful for the treatment and/or prevention of the digestive tract cancer. Further investigations are recommended.

## Figures and Tables

**Table 1:** Antiproliferative effects of compounds **1**–**4** from “tea flower” on human digestive tract carcinoma HSC-2, HSC-4, MKN-45, and Caco-2 cells.

Treatment	IC_50_ (µM) ^a^			
	HSC-2	HSC-4	MKN-45	Caco-2
Chakasaponin I (**1**)	4.6	17.5	16.8	30.2
Chakasaponin II (**2**)	14.1	18.2	17.3	40.6
Floratheasaponin A (**3**)	4.4	6.2	4.5	19.3
(–)-Epigallocatechin 3-*O*-gallate (**4**)	28.3	27.2	>100 (70.3)	>100 (71.8)
5-FU	>100 (55.5)	>100 (77.3)	>100 (52.4)	>100 (80.2)
Cisplatin	14.5	7.0	circa 100	>100 (69.5)
Doxorubicin	0.040	0.18	0.12	>100 (80.9)
Camptothecin	0.020	0.089	0.10	>100 (63.7)
Taxol	0.0012	0.0059	0.15	>100 (61.5)

Each value represents the mean ± S.E.M. (*n =* 4); ^a^ values in parentheses present percent of cell viability at 100 µM; commercial 5-FU (5-fluorouracil), cisplatin, doxorubicin, and camptothecin were purchased from Wako Pure Chemical Industries, Ltd. (Osaka, Japan) and taxol was from Tocris Bioscience (Bristol, UK).

**Table 2:** Effects of **1**–**4**, 5-FU, and cisplatin on cell cycle distribution of HSC-2 cells for 48 h.

Treatment	Concentration (µM)	Cell Cycle (%) ^a^		
		G0/G1 Phase	S Phase	G2/M Phase
Control	–	73.5 ± 0.8	10.8 ± 0.4	15.6 ± 0.7
Chakasaponin I (**1**)	5	71.6 ± 0.4	10.2 ± 0.4	18.0 ± 0.1
–	7.5	69.1 ± 1.4 *	10.6 ± 0.8	20.1 ± 0.8 **
–	10	59.9 ± 0.2 **	11.2 ± 1.0	28.7 ± 0.9 **
Control	–	72.4 ± 1.1	11.6 ± 0.4	16.0 ± 0.7
Chakasaponin II (**2**)	10	60.3 ± 0.1 **	10.5 ± 0.4	28.9 ± 0.3 **
–	12.5	44.1 ± 1.3 **	18.8 ± 2.4 **	36.8 ± 1.3 **
–	15	49.2 ± 0.8 **	20.0 ± 0.3 **	30.5 ± 0.6 **
Control	–	76.5 ± 1.4	9.8 ± 0.6	13.5 ± 0.8
Floratheasaponin A (**3**)	2.5	69.4 ± 1.0 *	11.8 ± 0.1	18.5 ± 0.9 *
–	5	67.6 ± 1.7 **	10.3 ± 0.7	21.7 ± 0.9 **
–	7.5	53.1 ± 0.8 **	15.9 ± 1.8 **	30.3 ± 1.1 **
Control	–	78.5 ± 1.2	9.4 ± 0.5	11.8 ± 0.7
(–)-Epigallocatechin 3-*O*-gallate (**4**)	25	78.8 ± 1.1	9.7 ± 0.5	11.2 ± 0.6
–	50	78.6 ± 1.2	9.0 ± 0.5	12.0 ± 0.6
–	75	76.8 ± 0.5	10.1 ± 0.1	12.7 ± 0.4
5-FU	30	36.4 ± 0.3 **	37.9 ± 0.9 **	25.4 ± 0.6 **
Cisplatin	3	50.7 ± 0.7 **	27.9 ± 0.5 **	20.0 ± 0.3 **

Each value represents the mean ± S.E.M. (*n =* 3); ^a^ cell cycle distribution was measured by Muse^®^ Cell Analyzer using a Muse Cell Cycle Assay Kit (Merck Millipore); asterisks denote significant differences from the control group, * *p* < 0.05, ** *p* < 0.01; commercial 5-FU and cisplatin were purchased from Wako Pure Chemical Industries, Ltd. (Osaka, Japan).

**Table 3:** Effects of **1**–**4** and camptothecin on the apoptosis of HSC-2 cells for 24 h.

Treatment	Concentration (µM)	Total Apoptotic Cells (%) ^a^
Control	–	8.9 ± 1.5
Chakasaponin I (**1**)	5	10.0 ± 0.4
–	7.5	35.6 ± 3.0 **
–	10	89.7 ± 0.4 **
Control	–	6.3 ± 0.4
Chakasaponin II (**2**)	10	7.0 ± 0.3
–	12.5	35.7 ± 0.4 **
–	15	64.2 ± 1.0 **
Control	–	12.9 ± 0.4
Floratheasaponin A (**3**)	2.5	17.5 ± 1.0
–	5	18.7 ± 0.3 *
–	7.5	50.1 ± 1.9 **
Control	–	13.9 ± 0.5
(–)-Epigallocatechin 3-*O*-gallate (**4**)	25	14.4 ± 0.6
–	50	13.1 ± 0.1
–	75	12.7 ± 0.1
Camptothecin	0.02	16.9 ± 0.2 **
–	0.05	43.9 ± 0.7 **

Each value represents the mean ± S.E.M. (*n =* 3); ^a^ cell cycle distribution was measured by Muse^®^ Cell Analyzer using a Muse Annexin-V and Deal Cell Kit (Merck Millipore); asterisks denote significant differences from the control group, * *p* < 0.05, ** *p* < 0.01; commercial camptothecin was purchased from Wako Pure Chemical Industries, Ltd. (Osaka, Japan).

**Table 4:** Effects of **1**–**4** and camptothecin on caspase-3/7 in HSC-2 cells.

Treatment	Concentration (µM)	Total Apoptotic Cells (%) ^a^
Control	–	7.1 ± 0.4
Chakasaponin I (**1**)	5	25.9 ± 3.8 **
–	7.5	59.6 ± 6.3 **
–	10	90.2 ± 0.9 **
Control	–	2.8 ± 0.1
Chakasaponin II (**2**)	10	5.9 ± 0.1
–	12.5	35.6 ± 1.5 **
–	15	50.9 ± 9.7 **
Control	–	4.2 ± 0.5
Floratheasaponin A (**3**)	2.5	27.9 ± 0.6 **
–	5	27.9 ± 1.0 **
–	7.5	45.3 ± 0.6 **
Control	–	7.2 ± 0.4
(–)-Epigallocatechin 3-*O*-gallate (**4**)	25	5.9 ± 0.2
–	50	4.9 ± 0.4
–	75	5.9 ± 0.1
Camptothecin	0.03	67.2 ± 1.2 **
–	0.05	75.0 ± 2.1 **

Each value represents the mean ± S.E.M. (*n =* 3); ^a^ cell cycle distribution was measured by Muse^®^ Cell Analyzer using a Muse Caspase-3/7 Kit (Merck Millipore); asterisks denote significant differences from the control group, ** *p* < 0.01; commercial camptothecin was purchased from Wako Pure Chemical Industries, Ltd. (Osaka, Japan).

## Supplementary Materials

Supplementary materials can be found at www.mdpi.com/1422-0067/17/12/1979/s1.
