# Supplementary material for: The Antiproliferative Effect of Chakasaponins I and II, Floratheasaponin A, and Epigallocatechin 3-O-Gallate Isolated from Camellia sinensis on Human Digestive Tract Carcinoma Cell Lines
Source: Int J Mol Sci. 2016 Nov 26;17(12):1979. doi: 10.3390/ijms17121979 (PMC5187779; doi:10.3390/ijms17121979)
Supplement: Supplementary file 1 [file ijms-17-01979-s001.pdf]

Int. J. Mol. Sci. **2016**, *17*, 1979; doi:10.3390/ijms17121979

Supplementary Materials: The Antiproliferative Effect of Chakasaponins I and II, Floratheasaponin A, and Epigallocatechin 3-*O*-Gallate Isolated from *Camellia sinensis* on Human Digestive Tract Carcinoma Cell Lines

Niichiro Kitagawa, Toshio Morikawa, Chiaki Motai, Kiyofumi Ninomiya, Shuhei Okugawa, Ayaka Nishida, Masayuki Yoshikawa and Osamu Muraoka

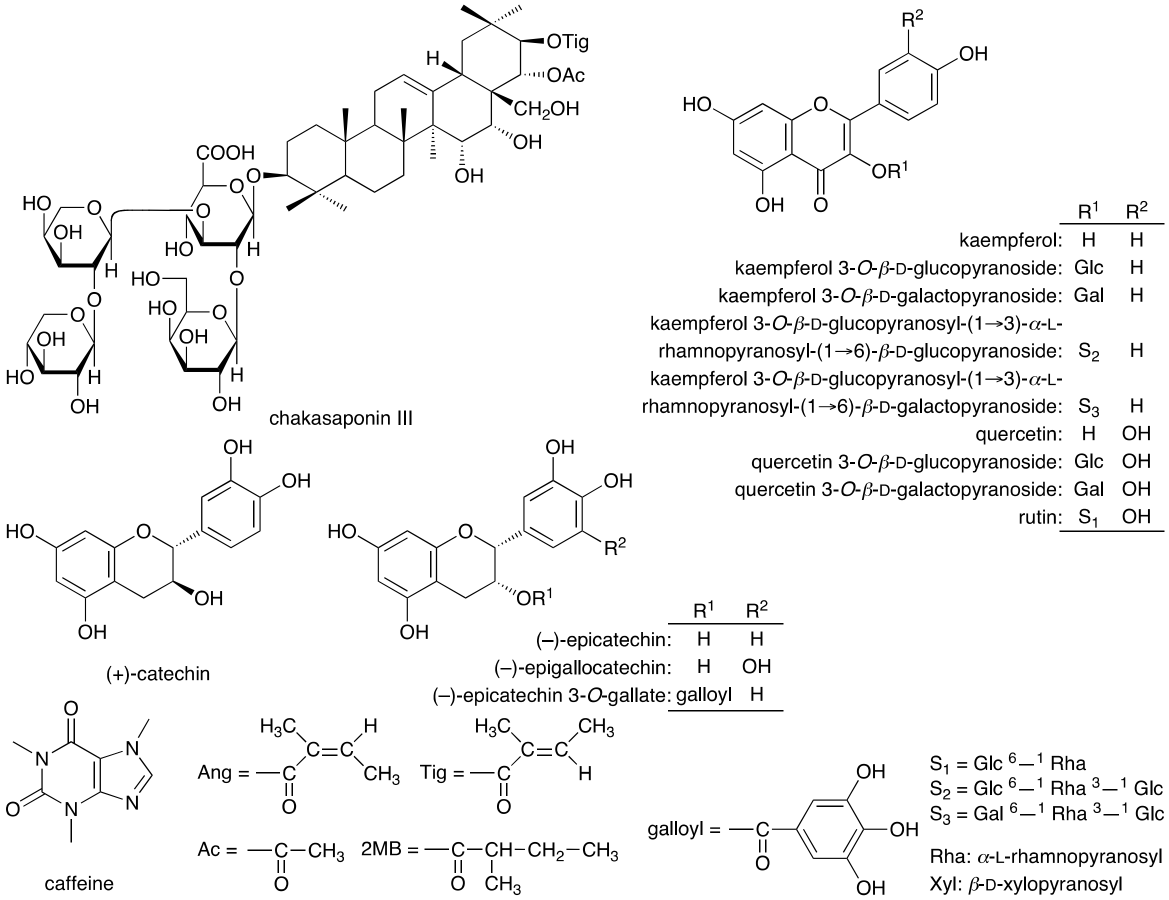

Figure S1. Chemical constituents from "tea flower".

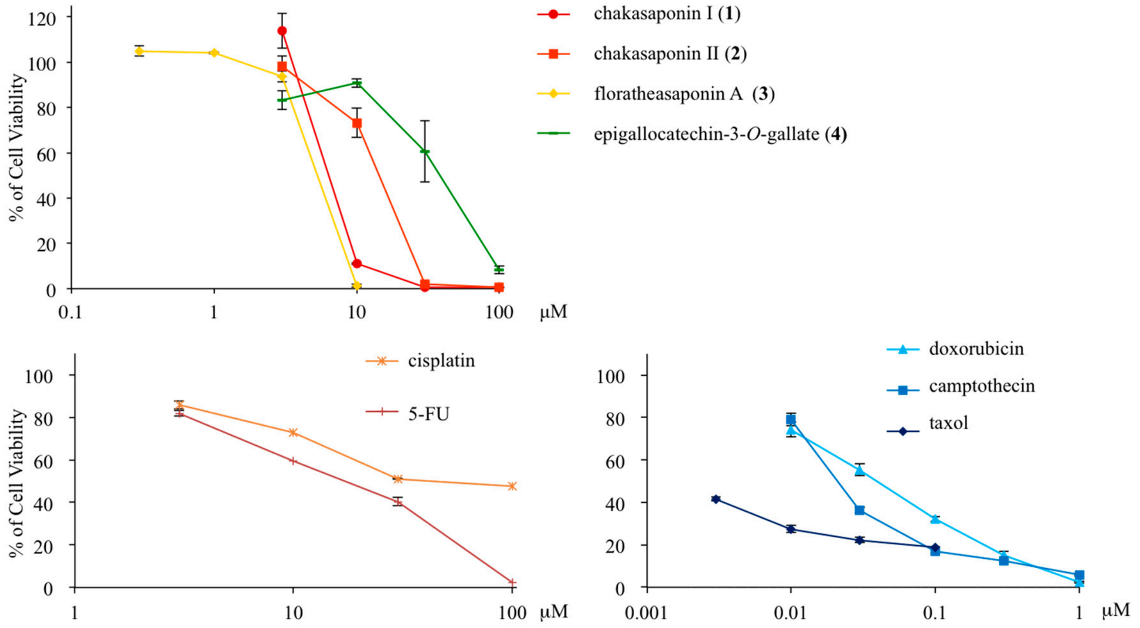

Figure S2. Concentration dependencies of antiproliferative activities of **1**–**4**, cisplatin, 5-FU, doxorubicin, camptothecin, and taxol against HSC-2 cells. Cell viability was determined by the MTT assay after incubation for 48 h with 3, 10, 30, and 100 μM of **1**, **2**, **4**, cisplatin, and 5-FU, 0.3, 1, 3, and 10 μM of **3**, and 0.01, 0.03, 0.1, 0.3, and 1 μM of doxorubicin and camptothecin, and 0.003, 0.01, 0.03, and 0.1 μM of taxol. The data represent the mean ± S.E.M. (*N* = 4); commercial cisplatin, 5-FU, doxorubicin, and camptothecin were purchased from Wako Pure Chemical Co., Ltd. (Osaka, Japan) and taxol was from Tocris Bioscience (Bristol, UK).

Table S1. Antiproliferative effects of constituents from "tea flower" on human digestive tract carcinoma HSC-2, HSC-4, MKN-45, and Caco-2 cells.

Treatment	IC_{50} (μM) [a]			
	**HSC-2**	**HSC-4**	**MKN-45**	**Caco-2**
Chakasaponin III	19.4	22.1	21.1	52.2
(+)-Catachin	>100 (115.3)	>100 (112.4)	>100 (89.3)	>100 (92.9)
(−)-Epicatachin	>100 (106.3)	>100 (129.8)	>100 (101.2)	>100 (94.8)
(−)-Epigallocatechin	54.6	23.8	ca. 100	>100 (83.9)
(−)-Epicatechin 3-*O*-gallate	>100 (67.2)	>100 (63.2)	>100 (73.9)	>100 (95.2)
Kaempferol	>100 (96.5)	>100 (118.8)	>100 (80.7)	>100 (105.1)
Kaempferol 3-*O*-Glc	>100 (122.1)	>100 (78.4)	>100 (118.5)	>100 (91.4)
Kaempferol 3-*O*-Gal	>100 (86.1)	>100 (111.0)	>100 (110.1)	>100 (100.0)
Kaempferol 3-*O*-Glc-(1→3)-Rha-(1→6)-Glc	>100 (107.6)	>100 (83.0)	>100 (95.7)	>100 (99.2)
Kaempferol 3-*O*-Glc-(1→3)-Rha-(1→6)-Gal	>100 (91.0)	>100 (82.4)	>100 (84.9)	>100 (88.3)
Quercetin	>100 (77.7)	77.2	>100 (52.0)	>100 (105.5)
Quercetin 3-*O*-Glc	>100 (56.5)	67.3	>100 (51.5)	>100 (110.5)
Quercetin 3-*O*-Gal	>100 (107.8)	>100 (93.8)	>100 (120.5)	>100 (93.5)
Rutin	>100 (119.9)	>100 (104.8)	>100 (86.7)	>100 (104.7)
Caffeine	>100 (98.3)	>100 (85.4)	>100 (85.0)	>100 (107.1)

Each value represents the mean ± S.E.M. (*n* = 4); [a] values in parentheses present percent of cell viability at 100 μM.
